# Focusing on NK cells and ADCC: A promising immunotherapy approach in targeted therapy for HER2-positive breast cancer

**DOI:** 10.3389/fimmu.2022.1083462

**Published:** 2022-12-19

**Authors:** Feifei Li, Sheng Liu

**Affiliations:** Longhua Hospital, Shanghai University of Traditional Chinese Medicine, Shanghai, China

**Keywords:** HER-2 positive breast cancer, NK cells, ADCC, immunotherapy approach, monoclonal antibodies

## Abstract

Human epidermal growth factor receptor 2 (HER2)-positive breast cancer has a high metastatic potential. Monoclonal antibodies (mAbs) that target HER2, such as trastuzumab and pertuzumab, are the cornerstone of adjuvant therapy for HER2-positive breast cancer. A growing body of preclinical and clinical evidence points to the importance of innate immunity mediated by antibody-dependent cellular cytotoxicity (ADCC) in the clinical effect of mAbs on the resulting anti-tumor response. In this review, we provide an overview of the role of natural killer (NK) cells and ADCC in targeted therapy of HER2-positive breast cancer, including the biological functions of NK cells and the role of NK cells and ADCC in anti-HER2 targeted drugs. We then discuss regulatory mechanisms and recent strategies to leverage our knowledge of NK cells and ADCC as an immunotherapy approach for HER2-positive breast cancer.

## Introduction

Human epidermal growth factor receptor 2 (HER2)-positive breast cancer accounts for 15%-20% of all types of breast cancer (1), and it is characterized by a high recurrence rate and poor prognosis (2). The targeted drugs for HER2-positive breast cancer include trastuzumab (3), pertuzumab (4), and trastuzumab emtansine (T-DM1) (5), which are the standard first- and second-line drugs. In addition, highly promising targeted drugs, such as magrolimab (6) and margetuximab (7), have significantly improved the survival rates of HER2-positive breast cancer patients. These monoclonal antibodies (mAbs) bind *via* the fragment crystalline (Fc) of immunoglobulin G1 (IgG1) to the Fcγ receptor III (FcγRIII) (CD16) on natural killer (NK) cells and elicit the release of cytotoxic factors, in a process known as antibody-dependent cell-mediated cytotoxicity (ADCC).

Immunotherapy has been established as a pillar of cancer treatment. In phase-I trial, involving 31 patients with refractory HER2-positive breast cancer, infusion with expanded activated autologous NK cells enhanced trastuzumab-mediated ADCC and produced a potent killing effect on breast cancer (8). The ADCC effect is an important mechanism in mAbs of HER2-positive breast cancer (9). ADCC has received accumulating attention given its substantial contribution to the therapeutic efficacy of mAbs. Understanding the role of ADCC in the immune response to mAbs will allow us to rationally combine these types of treatments in the context of HER2-positive breast cancer. In this paper, we summarize the advances of NK cells, ADCC effect, and corresponding research strategies in targeted therapy for HER2-positive breast cancer.

## NK cells biology

### Origin and ontogeny

NK cells are innate lymphoid-like cells that trigger an immune response to eliminate tumors through the secretion of cytokines and lytic granules (10). The development and maturation of NK cells constitute a stepwise process that can be divided into five stages (11). In the first stage, progenitors retain CD34 expression and acquire CD45RA and CD10; the second stage is marked by the absence of CD10 expression and the acquisition of CD117; the third stage is marked by the down-regulation of CD34 expression and the acquisition of lymphocyte function-associated antigen (LFA-1). The fourth and fifth stages involve mature NK cells, which include CD56^bright^ CD16^dim^ and CD56^dim^ CD16^bright^ subpopulations, respectively (12).

Mature NK cells migrate to the circulation and periphery to perform their functions. The CD56^bright^ CD16^dim^ subpopulation mainly distributes in peripheral tissues and secretes chemokines and cytokines (13). The CD56^dim^ CD16^bright^ subpopulation exerts its cytotoxic activity by secreting perforin and granzyme A/B (14). Except the circulating NK cells, tissue-resident NK subpopulations are present in various organs (15). In the tumor microenvironment (TME), tumor-associated fibroblasts and tumor-induced immunosuppressive cells also regulate NK cells and their anti-tumor activity (16).

### Function

NK cells play a key role in defending against tumor initiation and metastasis as the first line of immunity. NK cells are considered the most promising tumor-killing effector cells other than T cells because they are not limited by the major histocompatibility complex (MHC) (17). NK cells have a broad spectrum of tumor-killing effects, which include ADCC, release of perforin and granzyme A/B, factor-associated suicide (Fas) and Fas ligand (FasL) interaction, and cytokine secretion (18).

### FcγRs

FcγRs are expressed on NK cells, and they include FcγR I (CD64), FcγR IIa (CD32A), FcγR IIb (CD32B), FcγR IIc (CD32C), FcγR IIIa (CD16), and FcγR IIIb (CD16B). Low-affinity FcγRs are important mediators of ADCC function, and include two activating receptors, namely, FcγR IIIa and FcγR IIa, and the sole inhibitory receptor FcγR IIb (19). The ADCC activity of mAbs is largely dependent on the CD16 expressed by NK cells (20).

## NK cell-mediated ADCC in targeted drugs of HER2-positive breast cancer

### Trastuzumab

Trastuzumab is a humanized IgG-type mAb that specifically binds to the extracellular segment IV of the HER2 receptor (21), it blocks the formation of homodimers between HER2 and other HER family members (22). The immune system plays a key role in the anti-tumor effect of trastuzumab, which occurs through the binding of the HER2 receptor by the fragment antigen binding (Fab) and Fc to the CD16 of NK cells (23).

In HER2-positive breast cancer mice, tumor growth was almost completely inhibited by trastuzumab treatment (13/17); however, the tumor-suppressive capability of trastuzumab was significantly reduced in hormonal mice lacking FcγR III (1/15). Mice lacking FcγR IIB exhibited more ADCC; by contrast, trastuzumab failed to prevent tumor growth in mice lacking FcγR IIIa. These results suggest the significant contribution of FcγR IIIa-dependent mechanisms to the anti-tumor effects of cytotoxic antibodies (24). Duong MN observed that adipocytes promoted resistance to trastuzumab by secreting extracellular matrix components that formed a physical barrier between the tumor, antibodies, and immune cells. The physical barrier inhibited the lysis of NK cells, which resulted in the reduced sensitivity to trastuzumab-mediated ADCC in HER2-positive breast cancer (25).

NK cells are the main immune cell type that exerts the effects of ADCC (26). Arnould L (27) showed that patients with an objective response to trastuzumab had increased leukocyte infiltration and ADCC activity. Varchetta S (28) reported that the ADCC effect of trastuzumab was positively correlated with pathological remission rates in HER2-positive breast cancer patients, which suggested that enhancing the ADCC effect can optimize the efficacy of trastuzumab. Beano A conducted a clinical observation of 26 patients with metastatic HER2-positive breast cancer treated with trastuzumab. After six months of treatment, 17 patients were rated as responders and 9 as non-responders, in accordance with the response evaluation criteria in solid tumors. The results revealed that NK cell and ADCC activities were significantly higher in responders, and non-responders showed a lower NK cell activity. In addition, progression-free survival (PFS) significantly increased in patients with high levels of NK cell activity (29).

### Pertuzumab

Pertuzumab is a humanized IgG-type mAb that complements trastuzumab by blocking the heterodimeric activation pattern of HER2. Piccart M reported that the addition of pertuzumab to standard adjuvant therapy prolonged invasive disease-free survival of HER2-positive breast cancer patients with positive axillary lymph nodes (30). In the latest Chinese Society of Clinical Oncology guideline, the treatment of trastuzumab in combination with pertuzumab was recommended as the first-line option in targeted therapy. Both trastuzumab and pertuzumab exert anti-HER2 effect by inhibiting HER2 signaling and inducing ADCC activity (31, 32). Diessner J (33) discovered that tumor cell killing *via* ADCC increased when the triple combination of trastuzumab, pertuzumab, and NK cells was applied to HER2-positive breast cancer cells compared with the extent of ADCC induced by a single antibody. This study demonstrated the immunotherapeutic benefit achieved by the combined application of trastuzumab and pertuzumab.

### T-DM1

T-DM1 is an antibody-drug conjugate (ADC) with two core functional components: trastuzumab and a potent chemotherapeutic drug. Similar to a nuclear missile, ADC drugs combine the two components through a special linker to achieve a precise tumor-killing effect. T-DM1 has been approved by the United States Food and Drug Administration (FDA) for the treatment of HER2-positive metastatic breast cancer (34). HER2-DC1 comprises intratumoral multiepitope MHC class II HER2 peptide-pulsed type I polarized dendritic cells. Ramamoorthi G observed that HER2-DC1 combined with T-DM1 increased the levels of tumor-infiltrating CD4 and CD8 T, B, natural killer T, and NK cells in HER2 breast cancer-bearing mice and promoted complete tumor regression (35). ADCC effects of T-DM1 have been reported in other HER2-overexpressing cancers, such as lung and ovarian cancers. The activity of T-DM1 is superior to those of trastuzumab, pertuzumab, and their combination in high HER2/neu-expressing epithelial ovarian cancer (36). In HER2-overexpressing lung cancer cell lines, T-DM1 intervention overcame gefitinib resistance, and its ADCC effect was similar to that of trastuzumab in the presence of interleukin (IL)-2 activated NK cells (37).

### Margetuximab

As an Fc-modified chimeric mAb, margetuximab was designed to increase binding to CD16 and decrease binding to inhibitory FcγRIIB (CD32B), which resulted in the increased activation of ADCC and NK cells (38). In 2020, margetuximab has been approved in combination with chemotherapy for the treatment of patients with HER2-positive metastatic breast cancer (39). Rugo HS reported that margetuximab plus chemotherapy for HER2-positive advanced breast cancer had an acceptable safety and statistical improvement in PFS compared with trastuzumab plus chemotherapy (40). In addition, the safety and synergistic effects of margetuximab plus pembrolizumab were demonstrated in a single-arm IB-2 trial involving HER2-positive gastric cancer (41). These studies demonstrated that Fc-optimized anti-HER2 agents have desirable clinical futures.

### Small-molecule tyrosine kinase inhibitors

Small-molecule TKIs can inhibit kinase activity and downstream signaling by targeting the intracellular structural domain of HER2. Studies revealed that TKIs can modulate mAb-mediated ADCC responses. Lapatinib significantly increased membrane HER2 levels, and the combination with trastuzumab provided the largest ADCC response in HER2-low breast cancer models compared with afatinib and neratinib (42). Okita R (43) stated that malignant mesothelioma cells (MPM) pretreated with lapatinib bound more trastuzumab than untreated cells, which suggests that combinations of lapatinib and trastuzumab may be a promising strategy for MPM treatment. Cavazzoni A (44) explored erlotinib in combination with cetuximab or trastuzumab, and a significant effect on ADCC and inhibition of tumor growth were observed in the treatment of non-small-cell lung cancer (NSCLC).

## Regulatory mechanisms and research strategies of NK cells and ADCC

As previously mentioned, NK cell-mediated ADCC plays an important role in anti-HER2 therapy (45). However, the cytotoxicity of NK cells decreases with the altered activation receptor phenotype in breast cancer patients. Compared with healthy donors, NK cells express lower levels of NK cell p30-related protein (NKp30), NKp46, and NK cell group 2 member D (NKG2D) in breast cancer patients (46). Therefore, the enhancement of NK cells and their ADCC effect is an effective way to improve the efficacy and sensitivity of trastuzumab (47).

**Figure 1:**
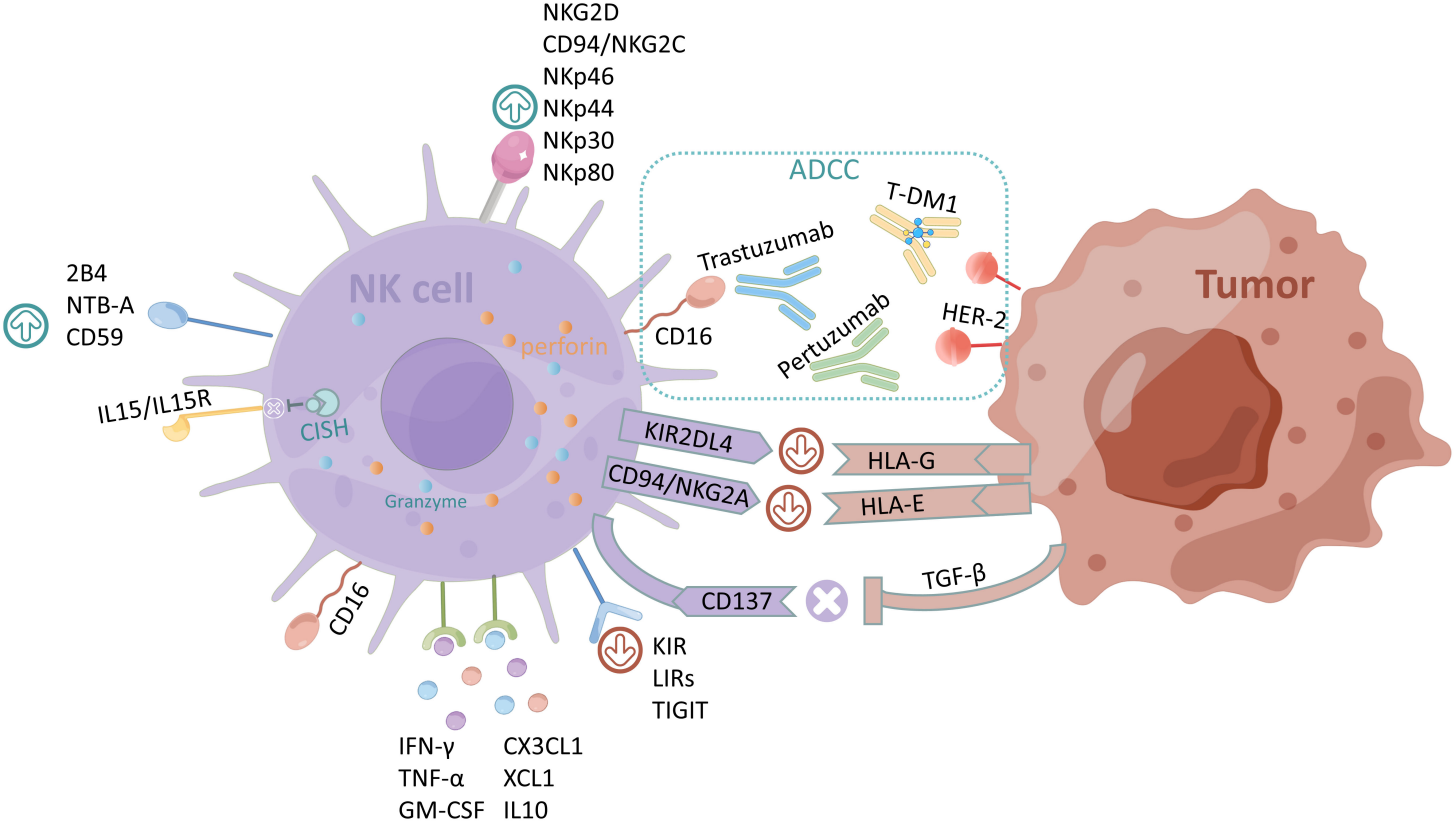
Regulatory mechanisms of NK cells and ADCC. The activating receptors of NK cells are NKG2D, CD94/NKG2C, NKp46, NKp44, NKp30, recombinant NK cell receptor 2B4 (NKR2B4, 2B4), NKp80, NTB-A, and CD59, and the inhibiting receptors are KIR, LIRs, and TIGIT. CISH normally inhibits IL-15/IL15R signaling in NK cells. The main cytokines secreted by NK cells are CX3CL1, chemokine (C motif) ligand 1 (XCL1), IL10, IFN-γ, TNF-α, and GM-CSF. HLA-G and HLA-E expressed by tumor cells bind to inhibitory receptors KIR2DL4 and CD94/NKG2A of NK cells respectively to exert immunosuppressive effects; CD137 counteracts the immunosuppression caused by the overexpression of TGF-β by tumor cells. The figure was drawn by Figdraw.

The regulation of the effect on NK cells and ADCC is maintained by a dynamic balance between activating and inhibiting receptors (48). **Figure 1** summarizes the regulatory mechanisms of NK cells and ADCC. The activation of NK cells is tightly regulated by the balance of signals from inhibitory and activating receptors, which is controlled by multiple lineage-encoded receptors that enable these cells to sense and respond rapidly to changes in their environment (49, 50). N-Glycosylation of FcγRIII also affects receptor interactions with IgG, which has led to studies of antibody engineering with greatly improved ADCC activities (51). Recombinant cytokines, agonists that promote activation receptors, and antibodies that block inhibitory receptors can be used to enhance ADCC (52, 53). Based on the regulatory mechanism of NK cells and ADCC, we summarized relevant research strategies, including targeted activation and inhibitory receptors, cytokine therapy, antibody engineering, and chimeric antigen receptor (CAR)-NK cells.

### Targeted activation receptors

NKG2D is a C-type lectin surface receptor, and binding to its ligand mediates the immune response of NK cells to tumors (54). The specific binding of antibodies to NKG2D and HER2 increases the cytotoxicity of peripheral blood mononuclear cells, thus significantly enhancing the killing activity of CAR-NK cells against the HER2-positive primary trastuzumab-resistant cell line JIMT-1 (55). Natural cytotoxicity receptors (NCRs) are prominent among activating NK cell receptors, and they are notably the only NK-activating receptors that can recognize pathogen-derived ligands. NCRs include the most specific NK cell markers, namely, NKp46 (NCR1, NCTR1, and CD335), NKp44 (NCR2, NCTR2, and CD336), and NKp30 (NCR3, NCTR3, and CD337), all three being members of the immunoglobulin superfamily (56).

NKp80 is a homodimeric C-type lectin-like receptor linked to the NKp80 locus gene, which is preferentially expressed by myeloid cells, thus promoting NK and myeloid intercellular crosstalk (57). Peipp M fused the extracellular structural domains of the ligands of NKp30 and NKp80 with single-chain fragment variables (scFv) targeting HER2 (named B7-H6:HER2-scFv and AICL:HER2-scFv, respectively). The results showed that antibody-derived proteins involved in NKp30 or NKp80 triggered NK cells to kill HER2-positive breast cancer. Moreover, the cytotoxicity of NK cells was synergistically enhanced when combined with the HER2-specific immune ligands of NKG2D (58). In addition, B7-H6:HER2-scFv and AICL:HER2-scFv synergistically enhanced the ADCC of the therapeutic antibodies trastuzumab and cetuximab, respectively. Kellner C constructed bispecific immunoligand UL16 binding protein 2 (ULBP2):HER2-scFvm, which can promote NK cell cytotoxicity against tumors and enhance ADCC in combination with cetuximab (59). The anti-CD137 agonist urelumab can overcome transforming growth factor (TGF)-β-mediated inhibition of human NK-cell proliferation and anti-tumor function and preserve the expressions of NKG2D, granzyme B, and interferon-gamma (IFN-γ) in HER2-positive primary breast cancer (60). Combined treatment with NKG2D agonist, 4-1BB antibody, and IL-27 improved activator receptor expression in NK cells and promoted the secretion of IFN-γ and tumor necrosis factor (TNF-α) while reducing the expression of the inhibitory receptor CD158a and the secretion of IL-10 in NK cells (61).

### Targeted inhibitory receptors

NK cells detect the absence of self-molecules on potential target cells through their inhibitory receptors, which is known as a recognition strategy of “loss of self.” The inhibitory receptors form three families, namely, killer cell immunoglobulin-like receptors (KIRs), leukocyte immunoglobulin-like receptors (LIRs), and NKG2A. KIRs and LIRs are members of the immunoglobulin superfamily and type I transmembrane molecules that recognize human leukocyte antigen (HLA) A, B, and C (HLA-I class a). LIRs mainly recognize non-classical HLA-G (class I b) molecules. NKG2A is a member of the NKG2 family, which belongs to the C-type lectin family of receptors and recognizes non-classical HLA-E class I molecules. KIRs/NKG2A plays a major role in mediating inhibitory signaling by binding to HLA (58).

Experimental and clinical studies on targeting inhibitory receptors are gradually being conducted. HLA-G can desensitize breast cancer cells to trastuzumab by binding to KIR2DL4, an atypical member of the KIR family, which responds to HLA-G *via* endosomal signaling (62). In HER2-positive breast cancer, blocking HLA-G/KIR2DL4 signaling improved trastuzumab resistance (63).

Monalizumab, a humanized anti-NKG2A mAb, is currently undergoing clinical trials in a variety of solid tumors (64). Frazao A showed that NK cells from tumor-draining lymph nodes expressed high NKG2A and checkpoint programmed cell death protein 1 (PD-1), which supported their potential as targets for immunotherapy using anti-NKG2A and/or anti-PD-1 in breast cancer (65). Phase II clinical trials suggest that monalizumab can enhance NK cell-mediated ADCC and improve objective remission rates in squamous cell carcinoma of the head and neck (SCCHN) (66). However, clinical trials on NKG2A mAbs for the treatment of breast cancer have not been reported. The marketing of NKG2A mAbs needs the support of larger clinical trials.

KIR2D antagonists (lirilumab) promote NK cell and granzyme B expression in a nuclear factor-κB-dependent manner in autologous cervical cancer cells (67). Lirilumab combined with rituximab can enhance NK cell-mediated rituximab-dependent cytotoxicity in KIR transgenic and homozygous mouse lymphoma models (68). In a phase II study of locally recurrent SCCHN, a 43% response rate to anti-PD-1, nabumab, and lirilumab pathology was observed with a favorable DFS and excellent 2-year overall survival compared with previously treated high-risk patients (69). Clinical trial results for KIR antagonists were mixed, with a KIR2D-specific mAb IPH2101 terminated early due to the lack of clinical efficacy in a single-arm phase II clinical trial for multiple myeloma (70). Carlsten M (71) analyzed the possible reasons for the failure of this clinical trial given that IPH2101 marginally augmented the antimyeloma cytotoxicity of remaining KIR2D^dull^ patient NK cells; the overall response was diminished by significant contraction and reduced function of KIR2D-expressing NK cells. At present, no clinical study has reported lirilumab for the treatment of breast cancer, and its therapeutic effects deserve further exploration.

### Cytokines therapy

The activity of NK cells is also influenced by various pro-inflammatory cytokines, including IL-15, IL-2, IFN-γ, TNF, granulocyte-macrophage colony-stimulating factor (GM-CSF), TGF-β1, and C-X3-C motif chemokine ligand 1 (CX3CL1) (72). **Table 1** summarizes the effects of cytokines on NK cells and ADCC effects.

**Table 1:** Effect of cytokines on NK cells and ADCC effects.

Cytokine	Function	Main mechanism	Ref.
IL2	Activation	IL2 ex vivo treatment of NK cells can restore the impairment of ADCC, concomitant to the normalization of the expression of CD16 zeta molecules. Serum cytokine profiling demonstrated an increase in IFN-γ induced protein 10. Gene expression analysis revealed significant changes in a highly restricted set of genes, including forkhead box P3, IL-2 receptor antagonist, and CISH.	(73, 74)
IL-15	Activation	IL15 is a novel cytokine that activates NK cells through components of the IL-2 receptor. IL-15 was associated with increased expression of NK cell activation markers NKp46 and NKG2D and increased NK cell isolated ADCC activity, whereas inhibitory receptors PD-1 and Tim3 were reduced.	(75, 76)
IL-2/IL-15	Activation	IL-2 combined with IL-15 enhanced the expression of NKG2D receptor to inhibit Wilms’ tumor *via* the mitogen-activated protein kinase (MAPK) signaling pathway.	(77)
CX3CL1	Activation	CX3CL1 overexpression recruited NK cells and increased NK cell-mediated cytotoxicity in HER-2 positive breast cancer.	(78)
IL-23	Activation	IL-23-induced NK cell activation and stimulated IFN-γ production by CD56^bright^ NK cells, which involved MEK1/MEK2, c-Jun N-terminal kinase, and phosphatidylinositol-3 kinase pathways.	(79)
IL-21	Activation	IL-21-treated NK cells secreted high levels of IFN-γ, which enhanced NK cell activation through extracellular signal-regulated kinase and signal transducer and activator of transcription (STAT) 1 signaling pathway, thus inhibiting tumor growth.	(80)
TGF-β	Inhibition	TGF-β inhibited the production of IFN-γ in human NK cells and ADCC, and these effects were mediated through SMAD3.	(81, 82)
IL-1R8	Inhibition	The high concentrations of IFN-γ, TNF-α, GM-CSF, CCL3, and CXCL8 release were observed in NK cells with IL-1R8 transient silencing by electroporated siRNA.	(83)
IL-12	Activation	IL-12 is an essential cytokine involved in the generation of memory-like NK cells, and IL-12 combined with anti-TGF-β can increase the maturation of tumor-associated NK cells.	(84)
CISH	Inhibition	CISH is a key negative regulator of IL-15 signaling in NK cells, and it inhibits anti-tumor activity of human NK cell.	(85)

The current research on cytokines therapy is focused on IL2 and IL15. Xiong Q constructed a membrane-bound IL-2 (mbIL-2) consisting of human IL-2 and human IL-2Rα joined by a classic linker. The novel mbIL-2 improved NK-92 cell persistence and enhanced its anti-tumor activity (86). Fujii R observed that the IL-15SA/IL-15RA complex can act as an inhibitor to block the inhibitory effects of TGF-β on the NK cell activation markers CD226, NKG2D, and NKp30 in breast, lung, and prostate cancers (87). IL-12 is an essential cytokine involved in the generation of memory-like NK cells, and IL-12 alone can sustain human primary NK cell survival without providing IL-2 or IL-15 but is insufficient to promote human NK cell proliferation (88). NK cells in breast tumors express high levels of NKG2A and low levels of NKp46, perforin, and granzyme B. One week of treatment with IL-12 and anti-TGF-β resulted in the increased maturation of tumor-associated NK cells (84). The combination of STING agonists with IL2/anti-PD-1 synergized to stimulate sustained granzyme and cytokine expression by lung-infiltrating NK cells in two spontaneously metastasizing orthotopic breast tumor models (89).

Cytokine-inducible SH2-containing protein (CIS; encoded by the gene CISH) is the first member identified in the suppressors of the cytokine signaling protein family. CISH is a key negative regulator of IL-15 signaling in NK cells, and it plays a key role in the regulation of human NK cell metabolic activity and thereby modulates anti-tumor activity (85). CISH deletion favors the accumulation of NK cells in primary breast cancer, thus optimizing NK cell-killing properties, and decreases T cell immune receptors with immunoglobulin and ITIM domain (TIGIT) immune checkpoint receptor expression (90). The activation of STAT3 helps tumor cells to evade NK cell-mediated immune surveillance, which is associated with high expression of tumor-promoting cytokines and growth factors, such as IL-10, TGF-β, and vascular endothelial growth factor-A (91).

Novel cytokine superagonists and lipid nanoparticles (LNPs) were designed to overcome the shortages of conventional cytokines, such as poor half-life, circulation instability, and dose-limiting toxicity. Jiangyin Lv combined IgG4 Fc segments, soluble IL-15Rα, and IL-15 (N72D) into a homodimeric IL-15 superagonist (F4RLI). F4RLI significantly stimulated the proliferation of human CD3^+^CD8^+^ T cells and NK cells *in vitro* (92). Jin-Qing Liu generated novel LNPs encapsulated with mRNA encoding cytokines (including IL-12, IL-27, and GM-CSF) that induced potent infiltration of immune effector cells, including IFN-γ and TNF-α producing NK and CD8+T cells into tumors, offering a new strategy for oncology immunotherapy (93).

In HER2-positive gastric cancer, IL2 treatment restored trastuzumab-mediated resistance to ADCC while restoring the expression of the CD16 zeta molecule, which may have implications for the treatment of HER2-positive breast cancer (94). Blockade with a specific KIR2DL-1,2/3 mAb reversed NK-cell inhibition and significantly enhanced degranulation and IFN-γ production of IL-2-preactivated NK-cells in the presence of primary glioblastoma (GBM) cells (95). In pancreatic cancer, the stimulator of interferon gene (STING)-IL35 axis in B cells reduced the proliferation of NK cells and attenuated the NK-driven anti-tumor response (96). The team of Landolina N (83) transiently silenced IL-1R8 by electroporated siRNA, and significantly higher concentrations of IFN-γ, TNFα, GM-CSF, CCL3, and CXCL8 release were observed in NK cells with silenced IL-1R8. Disruption of the IL6R/STAT3 axis and TIGIT in prostate cancer cells can increase the cytotoxicity of NK-92 cells by increasing FasL and granzyme A/B (97). In a xenograft mouse model of B-cell lymphoma, the addition of ALT-803 to anti-CD20 mAb treatment in conjunction with NK cells reduced the tumor burden and improved survival (98). Siebert N linked fibroblast activating protein alpha (FAP) and a mutant IL-2 variant (IL-2v) to form a FAP-IL-2v conjugate. They observed that FAP-IL-2v reduced tumor growth of high-risk neuroblastoma and improved survival, with increased numbers of NK and cytotoxic T cells (99). We have compiled research strategies on potential targets and cytokines that are currently being studied or in clinical trials (**Table 2**).

**Table 2:** Strategies for potential targets and cytokines to enhance NK cells and ADCC.

Target	Drug/ClinicalExperimental stage	Disease	Main mechanism	Ref.
**Potential targets**				
NKp30/NKp80	B7-H6: HER2-scFv and AICL: HER2-scFv	Breast cancer	Enhances ADCC by the therapeutic antibodies trastuzumab and cetuximab synergistically	(58)
NKG2D/4-1BB/IL-27	NKG2D agonist	Prostate cancer	Improves activator receptor expression in NK cells and promotes the secretion of IFN-γ and TNF-α	(61)
ULBP2	ULBP2:HER2-scFv	Breast cancer	Promotes NK cell cytotoxicity against tumors and enhances ADCC in combination with cetuximab	(59)
CD137	Urelumab	Breast cancer	Overcomes TGF-β-mediated inhibition of human NK-cell proliferation and preserves the expressions of NKG2D, Granzyme B, and IFN-γ	(60)
NKG2A	Monalizumab/UPSTREAM trial	Recurrent/metastatic SCCHN	Stable disease was observed in 6 patients (23%) with a median duration of 3.8 months (95% confidence interval: 2.7-NE).	(100)
NKG2A	Monalizumab/Phase II Study	Unresectable, Stage III NSCLC	Patients in the durvalumab plus monalizumab group had high objective remission rates and long PFS.	(101)
NKG2A	Monalizumab/Dose-Ranging and Cohort-Expansion Study	Recurrent gynecological malignancies	Intravenous monalizumab (10 mg/kg) treatment every 2 weeks was well tolerated in pretreated gynecological cancer patients. Short-term disease stabilization was observed.	(102)
NKG2A	Monalizumab+cetuximab/Phase II Study	SCCHN	The objective remission rate was 31%. The most common adverse events were fatigue (17%), fever (13%), and headache (10%).	(66)
KIR2D	Lirilumab/Phase II Study	Locally recurrent SCCHN	Adjuvant ivolumab and lirilumab were well tolerated, with a 43% pathologic response rate.	(69 **)**
KIR2D	IPH2101/Phase II Study	Multiple myeloma	The study was terminated due to the lack of patients meeting the defined primary objective (50% decline in M-protein).	(70)
Cytokines				
IL2	FAP-IL-2v conjugates	High-risk neuroblastoma	Reduced tumor growth and improved survival, with increased numbers of NK and cytotoxic T cells	(99 **)**
IL2	Membrane-bound IL-2	Leukemia	Improved persistence of NK-92 cells and enhanced their anti-tumor activity	(86)
STING/IL2/PD-1	STING agonist	Breast cancer and lung metastasis	Synergized to stimulate sustained granzyme and cytokine expression by lung-infiltrating NK cells	(89)
IL-15	IL-15SA/IL-15RA complex	Breast, prostate, and lung cancers	Blocked the inhibitory effects of TGF-β1 on NK cell activation markers CD226, NKG2D, NKp30 and granzyme B and perforin	(87)
IL15	N-803	Pediatric recurrent and/or metastatic osteosarcoma, neuroblastoma, and GBM multiforme	Increased the proliferative capacity of NK cells and was associated with increased phosphorylation of STAT3, STAT5, AKT, and p38 MAPK	(103)
IL-12/TGF-β	/	Breast cancer	Increased maturation of tumor-associated NK cells	(84)
CISH	/	Breast cancer	CISH deletion also favored NCR signaling and antitumor functions.	(90)
IL-12, IL-27 and GM-CSF	LNPs encapsulated	Melanoma	Induced potent infiltration of immune effector cells and increased secretion of IFN-γ and TNF-α	(93)
IgG4 Fc fragment and IL-15/IL-15Rα	F4RLI (homodimer IL-15 super agonist)	Colorectal cancer	Stimulated the proliferation of human CD3+CD8+ T cells NK cells *in vitro*, with improved half-life and strong anti-tumor activity	(92)
IL6R/STAT-3	IL6R/STAT-3 inhibitors	Prostate cancer	Decreased STAT-3 phosphorylation level and increased NKP46 expression, thus increasing cytotoxicity of NK-92 cells by increasing FasL, granzyme A, and granzyme B	(97)
VIII Factors	VIII-FcFVIII complex	Hemophilia A	Efficient active NK cells in a CD16-dependent manner, leading to IFN-γ secretion and release of cytolytic perforin and granzyme B	(104)
TNF	Recombinant Mouse IgG2a Antibody TA99	Melanoma	Promoted infiltration of NK cells and macrophages into B16 melanoma	(105)

### Antibody engineering and glycoengineering

**Figure 2:**
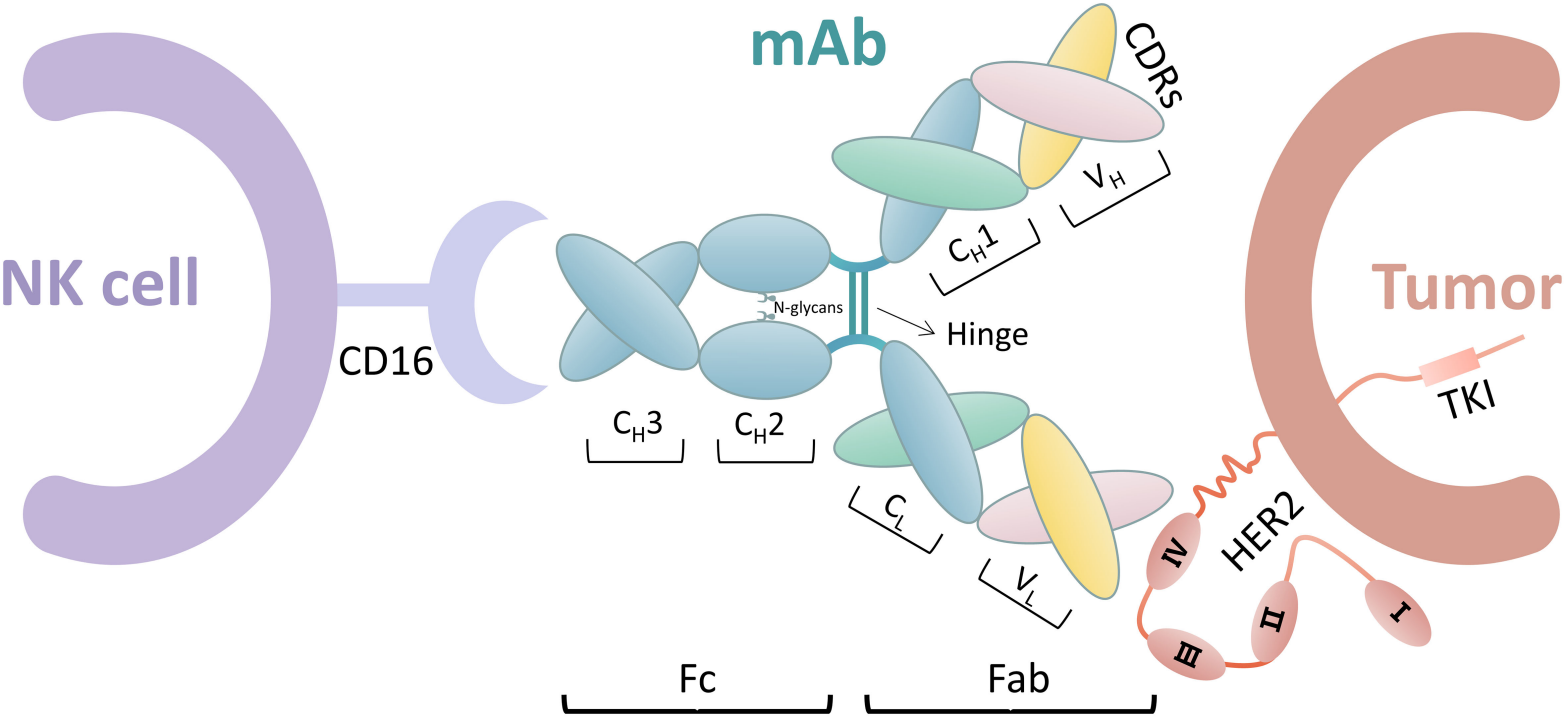
ADCC process including NK cells, mAb, and tumor cells. The mAbs include the Fab and Fc segments. The Fab segment is an endometrioid by heavy-chain constant region (C_H_1) and heavy chain variable region (V_H_), in which the amino acid sequence is constant. The amino acid sequences of light-chain constant region (C_L_) and heavy-chain variable region (V_L_) sites are variable. Complementary determining regions (CDRs) of the Fab segment bind on tumor cells by different amino acid sequences, such as trastuzumab binding to HER2 extracellular region IV. The Fc segment includes C_H_2 and C_H_3. NK cells bind to the Fc segment of mAb *via* CD16. CD16 interacts with C_H_2 of mAb in a 1:1 manner, where the N-glycan chain at the N297 position on C_H_2 also plays a key role in this interaction.

FcγR IIIa (CD16) binds to the Fc segment of IgG1 antibodies by interacting with the hinge region and methylene structural domain. This interaction is significantly influenced by the glycan of Asn 297 at the N-glycosylation site in each methylene structural domain. Therefore, studies have been conducted to enhance the interaction of the Fc segment with FcγR IIIa to improve the ADCC effect by techniques, such as glycoengineering of Fc N-glycans and design of Fc structural domains (106). **Figure 2** shows the ADCC process.

Hsiao H-C observed that hinge cleavage occurred when pertuzumab was incubated with cancer cells, which led to a substantial loss of ADCC (107). They constructed the protease-resistant version of the anti-hinged mAb to restore the ADCC. Kinder M demonstrated that discrete mutations in the CH2 region can compensate for the loss of function associated with mutation of the lower hinge of IgG1, thus initiating the complement cascade and facilitating potent ADCC (108). Oberg HH designed a trimeric structure of bsAb, that is, [(HER2)xCD16], which can redirect CD16-expressing γδ T cells in addition to NK cells to lyse HER2-expressing tumor cells. Their team discovered that trimeric bsAb with trastuzumab enhanced γδ T cell- and NK cell-mediated expression by an increased degranulation (109). Lin X designed a nano-immunomodulator AuNSP@αCD16, which exhibited high gene transfection efficiency and stable near infrared-II photothermal capacity  (110). AuNSP enables αCD16 gene transfection by modifying the tumor surface with CD16 antibodies, thus forming a tumor surface that allows NK cells to exert their recognition ability in the TME. This function increases the release of cytolytic particles and pro-inflammatory cytokines and ultimately enhances the killing function of NK cells against solid tumors.

Hong S observed that tumor cells upregulate sialylated glycans, which counteract NK-induced killing *via* the Siglec–sialylated glycan interaction. In addition, the high-affinity ligand of Siglec-7 leads to multifaceted consequences in the modulation of NK activation (111). The team of Hong S also reported a chemoenzymatic glycocalyx editing strategy to introduce high-affinity and specific CD22 ligands onto NK-92MI and cytokine-induced NK cells to achieve tumor-specific CD22 targeting (112). Madsen CB created glycoengineered breast cancer cells *via* zinc finger nuclease knockout (KO) of the core 1 enzyme chaperone (COSMC) for glycoengineering, and the ADCC effect mediated to NK cells increased in the COSMC KO breast cancer cell line (113).

### CAR-NK

CAR-T therapy has been approved by the FDA for the treatment of B-cell lymphoma and acute lymphoblastic leukemia. Compared with CAR-T therapy, CAR-NK therapy possesses unique advantages, such as the absence of autoimmune response and its independent killing capability (114). CAR-NK therapy has been carried out in a number of solid tumors in hematological diseases (115), breast cancer (116), and ovarian cancer (117).

Retargeting of NK cells with CARs or mAbs is a promising strategy to overcome tumor resistance. Eitler J reported that retargeting by CAR and/or the FcR/mAb (ADCC) axis provides the necessary signals for granule polarization and overcoming the resistance of HER2-positive breast tumors (118). Portillo AL demonstrated that HER2 CAR expression in NK cells enhanced the anti-tumor functions of patients with HER2-positive breast cancer, regardless of MHC class I expression (119). CAR-NK cells secreted IL-15 to maintain their anti-relapsed/refractory acute myeloid leukemia (AML) function and exhibited a high anti-AML activity. However, this benefit was transient due to the limited persistence of CAR-NK cells (120). Low-dose IL-2 was included in several clinical trials as a single treatment or as adjuvant therapy in combination with peripatetic NK and T cell transfer therapy (121). These studies showed that CAR-NK cells may be a highly potent and safe source of immunotherapy in the context of HER2-positive breast cancer.

### ADCC effective stage

The ADCC effective stage is mainly accomplished by the polarized release of cytotoxic mediators, which consists of key steps, such as reorganization and accumulation of filamentous actin on the immunological synapse (IS), receptor aggregation, polarization of the microtubule organizing center (MTOC) to the IS with lysis granules, and secretion of toxic granule contents at the IS (122, 123).

The F-actin network increases in specific regions of the IS, as observed by three-dimensional-structured illumination microscopy and two-color super-resolution imaging. When NK cells are activated, the F-actin network opens with a diameter of approximately 250–500 nm (124) to guide lysis granules along microtubules toward the MTOC; this step allows lysis granules to rest precisely around the area of MTOC polarization (125). Particle polarization and degranulation involve multiple intermediate steps, which culminate in the directed secretion of lysed particle contents at the IS. Trastuzumab-refractory patients had high serum levels of inflammatory protein chitinase 3-like 1 (CHI3L1), which prevented the correct polarization of the microtubule-organizing center along with lytic granules to the IS by hindering the receptor of advanced glycation end-products. CHI3L1 blockade was synergized with ADCC to cure mice with HER2-positive xenografts (9).

## Conclusion

As one of the main mechanisms of action of anti-HER2-targeted drugs, targeting NK cells and their ADCC action is an effective way to improve anti-HER2 efficacy. The development of novel drugs is often tested first in hematologic or highly aggressive metastatic tumors, whereas the research on HER2-positive breast cancer is relatively late. mAb drugs, which were developed to activate and inhibit receptors on NK cells, are still mostly in preclinical studies. In conclusion, in the TME, enhancement of NK cell activity and ADCC is a tumor immunotherapy strategy to improve the clinical efficacy of anti-HER2 mAbs. However, for the complex regulatory mechanism of NK cells and the multi-level ADCC effector process, considerable basic and clinical research needs the joint efforts of biologists, antibody engineering scientists, drug researchers, and clinical workers.

## Author contributions

SL designed the study. FL drafted the manuscript. Both authors contributed to the article and approved the submitted version.
